# Polygenic coronary artery disease association with brain atrophy in the cognitively impaired

**DOI:** 10.1093/braincomms/fcac314

**Published:** 2022-11-30

**Authors:** Eric de Silva, Carole H Sudre, Josephine Barnes, Marzia A Scelsi, Andre Altmann, for the Alzheimer’s Disease Neuroimaging Initiative

**Affiliations:** Centre for Medical Image Computing, University College London, London, UK; NIHR University College London Hospitals Biomedical Research Centre, London, UK; Centre for Medical Image Computing, University College London, London, UK; MRC Unit for Lifelong Health and Ageing, University College London, London, UK; School of Biomedical Engineering and Imaging Sciences, King’s College London, London, UK; Dementia Research Centre, UCL Queen Square Institute of Neurology, London, UK; Centre for Medical Image Computing, University College London, London, UK; Centre for Medical Image Computing, University College London, London, UK

**Keywords:** Alzheimer’s disease, coronary artery disease, brain atrophy, polygenic risk score, white-matter hyperintensities

## Abstract

While a number of low-frequency genetic variants of large effect size have been shown to underlie both cardiovascular disease and dementia, recent studies have highlighted the importance of common genetic variants of small effect size, which, in aggregate, are embodied by a polygenic risk score. We investigate the effect of polygenic risk for coronary artery disease on brain atrophy in Alzheimer’s disease using whole-brain volume and put our findings in context with the polygenic risk for Alzheimer’s disease and presumed small vessel disease as quantified by white-matter hyperintensities. We use 730 subjects from the Alzheimer’s disease neuroimaging initiative database to investigate polygenic risk score effects (beyond *APOE*) on whole-brain volumes, total and regional white-matter hyperintensities and amyloid beta across diagnostic groups. In a subset of these subjects (*N* = 602), we utilized longitudinal changes in whole-brain volume over 24 months using the boundary shift integral approach. Linear regression and linear mixed-effects models were used to investigate the effect of white-matter hyperintensities at baseline as well as Alzheimer’s disease-polygenic risk score and coronary artery disease-polygenic risk score on whole-brain atrophy and whole-brain atrophy acceleration, respectively. All genetic associations were examined under the oligogenic (*P* = 1e-5) and the more variant-inclusive polygenic (*P* = 0.5) scenarios. Results suggest no evidence for a link between the polygenic risk score and markers of Alzheimer’s disease pathology at baseline (when stratified by diagnostic group). However, both Alzheimer’s disease-polygenic risk score and coronary artery disease-polygenic risk score were associated with longitudinal decline in whole-brain volume (Alzheimer’s disease-polygenic risk score *t* = 3.3, *P*_FDR_ = 0.007 over 24 months in healthy controls) and surprisingly, under certain conditions, whole-brain volume atrophy is statistically more correlated with cardiac polygenic risk score than Alzheimer’s disease-polygenic risk score (coronary artery disease-polygenic risk score *t* = 2.1, *P*_FDR_ = 0.04 over 24 months in the mild cognitive impairment group). Further, in our regional analysis of white-matter hyperintensities, Alzheimer’s disease-polygenic risk score beyond *APOE* is predictive of white-matter volume in the occipital lobe in Alzheimer’s disease subjects in the polygenic regime. Finally, the *rate of change* of brain volume (or *atrophy acceleration*) may be sensitive to Alzheimer’s disease-polygenic risk beyond *APOE* in healthy individuals (*t* = 2, *P* = 0.04). For subjects with mild cognitive impairment, beyond *APOE*, a more inclusive polygenic risk score including more variants, shows coronary artery disease-polygenic risk score to be more predictive of whole-brain volume atrophy, than an oligogenic approach including fewer larger effect size variants.

## Graphical Abstract

**Graphical abstract:**
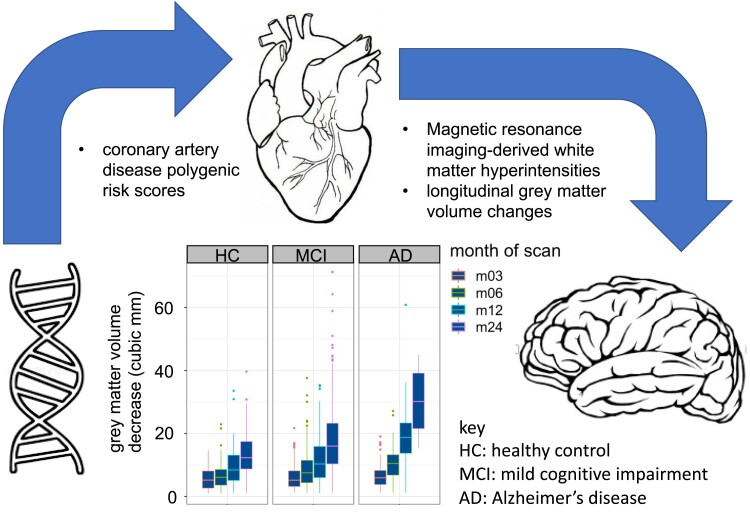

## Teaser

de Silva *et al*. report that in subjects with mild cognitive impairment, a coronary artery disease-polygenic risk score is more strongly associated with whole-brain volume atrophy than an Alzheimer’s disease-polygenic risk (excluding the *APOE* region). This highlights the importance of cardiac genetics and health in dementia.

## Introduction

It is estimated that more than 1 million people in the UK and over 44 million individuals globally^1^ are living with dementia. Alzheimer’s disease is the most common form of dementia and is usually diagnosed in the elderly (over the age of 65 years). There are many symptoms associated with Alzheimer’s disease and these include changes in memory, language and personality.^2^

Beyond cognitive testing, there has been a large focus on the use of brain imaging to help diagnose and track the disease. In particular, a decrease in brain volume and the build-up of protein in the form of amyloid plaques (between neurons) and misfolded neurofibrillary tangles of hyperphosphorylated tau (within neurons) are seen in brain imaging studies and observed in post-mortem examinations of Alzheimer’s disease patients.^3^ It is thought that the amyloid and tau aggregations contribute to the death of neuronal cells, resulting in a reduction in regional grey-matter volume and neuronal connectivity.^4^ Surprisingly, years of clinical trials involving pharmacological interventions targeting these protein deposits have largely been unsuccessful,^5^ possibly because they are formed as a result of earlier pathological changes. Another explanation may be that drug interventions, thus far, have all been administered too late in the disease life-course to be effective and that intervention earlier in life may be required. Current symptomatic treatments for Alzheimer’s disease symptoms come from acetylcholinesterase inhibitors and glutamate blockers, which serve, in some cases, to reduce disease severity, but are by no means curative.^6^ At the time of writing, the monoclonal antibody aducanumab, which targets beta-amyloid plaques, had just been approved for clinical use in the USA (see www.fda.gov).

Alzheimer’s disease is often diagnosed alongside vascular dementia in what is called mixed dementia.^7^ Vascular dementia (itself the second most common form of dementia following Alzheimer’s disease) is probably the result of reduced blood flow to brain cells, resulting in cell death.^8^ This can be caused by cerebral small vessel disease, resulting in subcortical vascular dementia, which affects vessels deep in the brain. One imaging marker of small vessel disease is magnetic resonance imaging (MRI)—visible white-matter changes, or, white-matter hyperintensities (WMHs). WMHs are known to increase with age,^9^,^10^ but are also strongly associated with Alzheimer’s disease.^11^ WMHs have also been associated with a range of other pathologies, including amyloid angiopathy, arteriosclerosis, axonal loss, blood–brain barrier leakage, demyelination, gliosis, hypoperfusion, hypoxia, and inflammation.^12^

In coronary artery disease (CAD), plaques aggregate in the blood vessels that feed the heart oxygen and nutrients. In the extreme, this can lead to angina and a heart attack, but a smaller, prolonged reduction in cardiac function may be responsible for cerebral hypoperfusion.^13^ CAD has a strong genetic basis, being ∼50% heritable with ∼60 genetic loci identified.^14^ However, the relationship between the genetic contribution to heart health and Alzheimer’s disease remains largely unexplored.

While age is the most salient factor in Alzheimer’s disease risk, there is, alongside environmental and lifestyle factors,^15^ a genetic component underlying both Alzheimer’s disease and CAD. In Alzheimer’s disease, a small number of cases (<5%) are due to autosomal dominant early-onset Alzheimer’s disease, for which there are a number of rare, large-effect-size genetic variants that contribute to the pathology.^16^ These include mutations that result in abnormal protein products of amyloid precursor protein, or in the genes that code for the enzymes that alter the breakdown of amyloid precursor protein, both of which may result in an increase in amyloid plaques.

Most Alzheimer’s disease cases are sporadic and late-onset (typically found in those 65 and older), where heritability is estimated to be between 60 and 80%.^17^ There are, a number of identified common variants, most notably the e4 allele of the *APOE* (apolipoprotein) gene, which accounts for ∼5% of Alzheimer’s disease heritability, plus about 20 additional loci that account for up to 30% Alzheimer’s disease heritability.^18^ It is likely that the remaining heritability is the result of the combination of a great many (1000s to 100 000s) common variants, each contributing a very small effect. Other studies^19^ argue that known loci account for a much smaller percentage of heritability and that many rare variants are each making small contributions, something only larger studies can address.

As a result of these findings, much research has been performed to establish how to best capture a composite measure of these many common variants that individually have such a small effect. Polygenic risk scores (PRSs) offer a way of doing this and have become increasingly used following the many large genome-wide association studies (GWAS), which show associations between common genetic variants and diseases. The PRS sums up the effect size^20^ across a selected set of genetic variants shared between a discovery sample (for example some CAD-GWAS) and target sample (some other genotyped cohort such as the Alzheimer’s disease neuroimaging initiative, ADNI), resulting in an aggregate score that reflects the genetic contribution to the disease phenotype in the target cohort. Alzheimer’s disease-PRS (AD-PRS) over varying *P*-value selection thresholds has effectively discriminated between Alzheimer’s disease cases and controls,^21^ been used as a predictor of conversion from mild cognitive impairment (MCI) to Alzheimer’s disease,^22^ been linked to inflammatory biomarkers,^23^ CSF amyloid beta levels,^24^ CSF tau levels,^25^ hippocampal volume,^26^ cortical thickness^27^ and age of onset of Alzheimer’s disease.^28^ AD-PRS-related work has been recently reviewed by.^29^

Just as research has been devoted to investigating how AD-PRS affects Alzheimer’s disease phenotypes, the same concept can be used to investigate genetic risk for other diseases on Alzheimer’s disease pathology. Here, we specifically investigate the role of common, small effect, cardiac-related genetic variants in Alzheimer’s disease. This enables us to elucidate the role that cardiovascular health plays in relation to dementia. While we focus here on the influence of underlying genetics upon WMHs, whole-brain atrophy and the changes therein, the cardiac-cerebrovascular axis is no doubt complex, encompassing many biological pathways. We believe, however, that a combination of genetics and imaging, in particular longitudinal images that capture changes over a disease trajectory, will provide important insights into this system.

## Materials and methods

Data used in the preparation of this article were obtained from the ADNI database (adni.loni.usc.edu). The ADNI was launched in 2003 as a public–private partnership, led by Principal Investigator Michael W. Weiner, MD. The primary goal of ADNI has been to test whether serial MRI, positron-emission tomography, other biological markers and clinical and neuropsychological assessment can be combined to measure the progression of MCI and early Alzheimer’s disease. For up-to-date information, see www.adni-info.org. The R-data-package ADNIMERGE (dated 19 May 2020) was used to access ADNI data.

### Computing white-matter hyperintensities

Regional and total WMH values were determined using the Bayesian Model Selection (BaMoS) software,^30^ a white-matter lesion segmentation algorithm. BaMoS was applied to 932 ADNIGO and ADNI2 participants following Walsh *et al.*^31^ and described therein. In short, the label fusion algorithm geodesic information flows^32^ was used to parcellate the T_1_-weighted cortical grey matter into various cortical and subcortical brain structures in an automated fashion. It additionally carries out skull stripping and generates probabilistic atlases for each individual. These atlases are then input to BaMoS alongside co-registered fluid attenuated inversion recovery images consisting of log-transformed normalized intensities. To determine white-matter lesions, BaMoS computes the most suitable model description of the data, accounting for prevailing outliers. It manages this by first partitioning the data into inlier and outlier portions and then modelling the input data in a hierarchical fashion, with elements then separated into one of four tissue types—grey matter, white matter, cerebrospinal fluid and non-brain. Each of these in turn is modelled via a Gaussian mixture model, with the number of constituent parts determined using a split-and-merge strategy. An expectation-maximization algorithm is used for optimization with model selection implemented using Bayesian information criterion to produce probabilistic lesion maps from which measurements of lesion volumes are inferred. Regional volumes in cubic millimetre were also computed across five lobes and four radial layers.

### Brain atrophy

To compute changes in brain volume, we utilize the boundary shift integral (BSI) method.^33,34^ Briefly, the BSI is defined as the difference in brain volume (either in total brain or in a brain region via displacement of the boundaries), automatically computed (brain mask creation is semi-automated) between a baseline scan and a repeat scan at a later time. The KN-BSI^34^ is an extension of the classic-BSI and carries out tissue-specific intensity normalization, which deals with tissue-contrast differences, producing a smaller standard deviation in atrophy changes than the classic-BSI. BSI values were obtained from the *foxlabbsi* table in ADNI, comprising 2348 subjects (across ADNI1, ADNI2, ADNI3 and ADNIGO), where all T_1_-weighted scans included in the core data sets pertaining to BSI were obtained using 3T scanners. Some months had very few available scans due to subject availability, so these were removed to maintain consistency between scan interval times. MRI scans were originally made using accelerated and non-accelerated acquisitions and we have chosen to focus on the accelerated scans^35^ as they have been shown to result in fewer motion artefacts in pairs of scans resulting from patient movement. Finally, some scans had BSI-determined brain volume increases, which may be a result of better subject hydration or due to overall noise. Scans with BSI < 0 [corresponding to an increase in whole-brain volume (WBV) over time] were removed from the analysis.

### ADNI genetic target data pre-processing

The genetic data used in this work is a combination of ADNI1, ADNIGO and ADNI2 participant genotypes, comprising a total of 1674 subjects.^36^ Details on quality control and imputation (use of the haplotype reference consortium reference panel, the Sanger server, EAGLE2 for phasing, and positional burrows wheeler transform for imputation) are described in Scelsi *et al*.^37^ Briefly, following imputation, multi-allelic single-nucleotide polymorphisms (SNPs) and SNPs with INFO scores <0.3 were removed; then calls with < 90% posterior probability of the imputed genotype were set to missing. SNPs missing in > 10% subjects, deviating from Hardy–Weinberg equilibrium (*P* < 5e-7) and with minor allele frequency <5% were all removed. These processing steps were carried out using PLINK v1.9^38^ (www.cog-genomics.org/plink/1.9/) and resulted in a final set of 5 082 879 autosomal SNPs. For ancestry determination and relatedness analysis, following Altmann *et al.* and Scelsi *et al.*^25,37^ a HapMap 3 reference panel was utilized where individuals with >80% central European ancestry were held. PLINK v1.9 was then used to retain common SNPs with minor allele frequency (MAF) ≥5% and carry out linkage disequilibrium (LD)-pruning and construct a genetic relatedness matrix (threshold = 0.1) and filter to remove related subjects. This resulted in the exclusion of 116 subjects, most likely due to their being genetically non-Central European or being related to other subjects.

For the CSF amyloid beta measurements we used ADNIMERGE *ABETA.bl* values, removed subjects with missing data and set values recorded as <1700 to 1700 (positron-emission tomography CSF Ab1-42; 192 pg/ml cut-off value; Luminex assay; data range 203–1700 with mean = 1024.7).

### Coronary artery disease and Alzheimer’s disease discovery GWASs

To investigate the PRS contribution due to Alzheimer’s disease, we utilized the largest currently available meta-GWAS of Alzheimer’s disease, featuring 35 274 clinical and autopsy-documented Alzheimer’s disease cases and 59 163 controls.^39^ Summary statistics were downloaded from the National Institute on Aging Genetics of Alzheimer’s Disease Data Storage Site (NIAGADS; July 2020), comprising 11 480 633 SNPs.

In order to investigate the PRS contribution due to CAD, we used a meta-analysis of 60 801 CAD cases and 123 504 controls.^40^ Summary statistics were downloaded from the CARDIoGRAMplusC4D [Coronary Artery Disease Genome-wide Replication and Meta-analysis plus the Coronary Artery Disease (C4D) Genetics] consortium (http://www.cardiogramplusc4d.org/data-downloads/) in July 2020, comprising 8 624 384 variants.

Given the very large effect size of *APOE*-e4 upon Alzheimer’s disease pathology, we removed the *APOE* region so as to explore genetic effects beyond this risk factor. The block removed from chromosome 19 (hg19 coordinates) comprises SNPs 44 400 375 (rs430308) to 46 500 052 (rs62113435).

### Polygenic risk scores

The PRS is a weighted sum of allele counts, where the weights are odds ratios (effect sizes) from a discovery GWAS and represent the strength of the association between the variant and the trait it is associated with. PRSs were computed using PRSice v2.1.9.^41^ To ensure that loci are isolated and independent, LD clumping was applied so that SNPs in LD with one another are removed such that the SNP with the lowest *P*-value within each LD block of correlated SNPs is held for analysis. LD clumping was conducted with a clumping window of 250 kb on either side of the index SNP, with an *r*^2^ threshold of 0.1 and a *P*-value threshold of 1.

The PRS for each subject was computed using the ‘–score avg’ setting in PRSice by summing up the product of each variant by the number of effective alleles observed divided by the number of alleles included for that individual. This last divisor makes the PRS scores more comparable between subjects in the presence of missing SNPs. PRS was calculated for each subject at two *P*-value cut-offs *P* = 1e-5 (i.e., genome-wide suggestive loci) capturing oligogenic effects and *P* = 0.5 capturing polygenic effects, which have been identified as sufficient to encompass threshold variation.^21^

Of note, it is imperative when working with PRS that there is no sample overlap between the discovery GWAS (Kunkle *et al*.^39^ ) and the target cohort (here the combined ADNI1, ADNIGO and ADNI2). We did not encounter any sample overlap as the discovery GWAS used the Alzheimer’s disease genetics consortium (ADGC) summary statistics, which only use healthy controls (HCs) and Alzheimer’s disease cases from ADNI1 comprising 1.5T scans. However, from the 846 individuals with imaging and genetic data, we excluded 116 subjects with non-Central European ancestry, resulting in a final sample size of 730 for our study. Moreover, demographic genetic differences arising from ancestral population structure have been found to bias PRS scores.^42^ This is the case particularly for high *P*-value cut-offs (e.g. *P* = 0.5) leading to PRS with many thousands of SNPs. To account for this effect, our linear regression models include the first five principal components of population structure as covariates.

### Second-order grey-matter changes

By plotting longitudinal WBV change or BSI (from baseline in units of cubic cm or ml, where 1 cm^3^ = 1 ml), for each subject taken at 3, 6, 12 and 24 months post-baseline, the gradient of the line of best fit gives a second-order change (the second derivative) in WBV or the rate of change of BSI per subject. We term this rate of change the ‘atrophy acceleration’, which provides a measure of how fast the WBV is declining. By way of illustration, if, for example, there is a BSI of 6 ml between Months 0–24, then the gradient would be 0.25 ml/month^2^ (which equals 3 ml/year^2^) and would have an intercept of zero.

### Statistical analysis

We first investigated PRSs for AD-PRS as well as PRSs for CAD (CAD-PRS) and their relationship with total WBV over a range of time points [which are normalized by dividing by intracranial volume (ICV)], total WMHs (total) at baseline (which are log-transformed) and CSF amyloid beta measurements. We used linear regressions within each diagnostic group, adjusting for age, sex, education, *APOE*-e4 burden (number of e4 alleles) and the first five principal components of population structure. In the case of the WMH (total), we also included the ICV as an additional covariate. Regressions provided *t*-values as well as *P*-values for the association between PRS and biomarkers; given the large number of comparisons (36 = 2 PRS thresholds × 2 PRS × 3 diagnoses × 3 biomarkers), we have adjusted *P*-values for multiple comparisons using a false discovery rate correction (rate = 5%). Given that both increasing whole-brain atrophy, WMHs as well as BSI values are known to lead to worse health outcomes, all *P*-values are based on one-tailed tests. For the regional WMH data, we carried out an identical analysis but over five lobar regions: frontal, parietal, occipital, temporal and combined basal ganglia, thalami and infratentorial volume. Each of these regions was normalized by its corresponding regional volume.

In addition, for the *atrophy accelerations*, we also used linear mixed-effects models (with subject as a random intercept and time since baseline, for brevity referred to as just time, as a random slope across diagnostic groups, which allowed us to capture the non-linear effects of the disease; R library lme4) across both *P*-value and PRS thresholds. Here subject is the random effect, so the atrophy acceleration is calculated per subject as above. In this way, we allow for a positive correlation among measurements for the same individual once the fixed effects have been accounted for. The number of months since the baseline is also included as a random effect. In order to get the influence of PRS on the ‘rate of atrophy’ (atrophy acceleration) the model includes the PRS-by-time interaction (PRS*time). This is all implemented using the R library function lme4 with the dependent variable being the BSI measurement and the independent variable being PRS*time or polygenic score-by-time interaction (where time is the months since baseline), across both *P*-value and PRS thresholds:

$$\begin{array}{rl} full\_model\;= & lmer(atrophy∼PRS^{*}time\;+\;AGE\;+\;SEX\\ & +\;EDUCATION\;+\;APOE4\;+\;PC1\ldots PC5\\ & +\;(1\;+\;time|ID)) \end{array}$$

Similar covariates as used in previous linear regressions and with the PRS*time comprise the fixed effects. We used a likelihood ratio test to compare full models and reduced models (without the polygenic score-by-time interaction in the reduced model) to compute model *P*-values as our hypothesis is that the time-by-PRS interaction is of interest:

$$\begin{array}{c} \begin{array}{rl} reduced\_model\;= & lmer(atrophy∼PRS\;+\;time\;+\;AGE\;+\;SEX\\ & +\;EDUCATION\;+\;APOE4\;+\;PC1\ldots PC5\\ & +\;(1\;+\;time|ID)) \end{array}\\ anova(full\_model,reduced\_model) \end{array}$$

## Results

### Filtering core data sets

**Figure 1:**
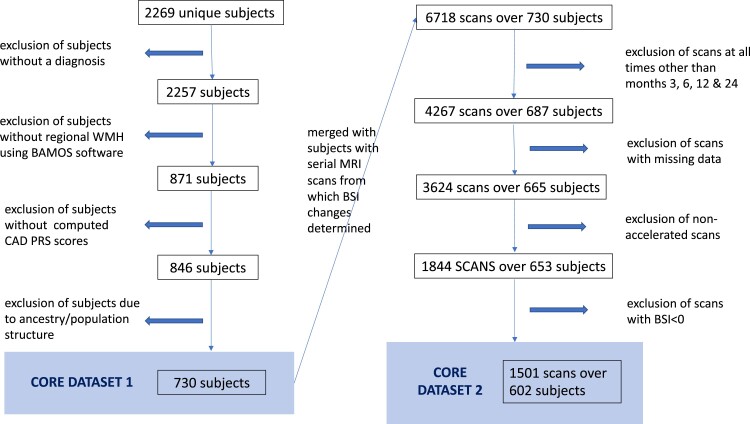
**Illustration of the selection of subjects filtered for analysis.** A total of 730 subjects comprise Core Data Set 1 and 602 subjects comprise Core Data Set 2.

A total of 2269 unique subjects were extracted from ADNIMERGE, of which 2257 had available baseline diagnoses. A subset of these subjects, with computed total and regional WMH using BaMoS (see below), results in a cohort of 871 individuals. Further subjects were excluded following the addition of AD-PRS and later CAD-PRS (134 456 and 135 584 variants, respectively) and principal components of population structure, leaving *N* = 730 subjects that comprise Core Data Set 1 (Fig. 1). To maximize the numbers per diagnostic group, the following subdiagnostic groups were merged: (i) control/normal (*N* = 129) and significant memory concern (*N* = 89) to ‘HC’ (*N* = 218); (ii) early MCI (*N* = 253) and late MCI (*N* = 137) to ‘MCI’ (*N* = 390); (iii) Alzheimer’s disease (*N* = 122) remaining unchanged.

We augmented Core Data Set 1 with available BSI data (ADNIMERGE *foxlabbsi* table) to investigate atrophy accelerations, i.e. changes in whole-brain volumes over time. Given the paucity of scans at Months 8, 18, 36, 48, 60, 72, 84, 96, 120, 132, 144 and 156 (Supplementary Fig. 2), only scans from Months 3, 6, 12 and 24 were retained (Supplementary Fig. 3). The remaining atrophy values comprised 824 instances of negative total KN-BSIs (across all months), implying growth in WBV over time. As this is unlikely, these scans were considered to be inaccurate and removed from the sample, which resulted in the loss of 51 subjects. This formed Core Data Set 2, which comprises 1501 scans over *N* = 602 subjects (Fig. 1).

### Core data set summaries

**Figure 2:**
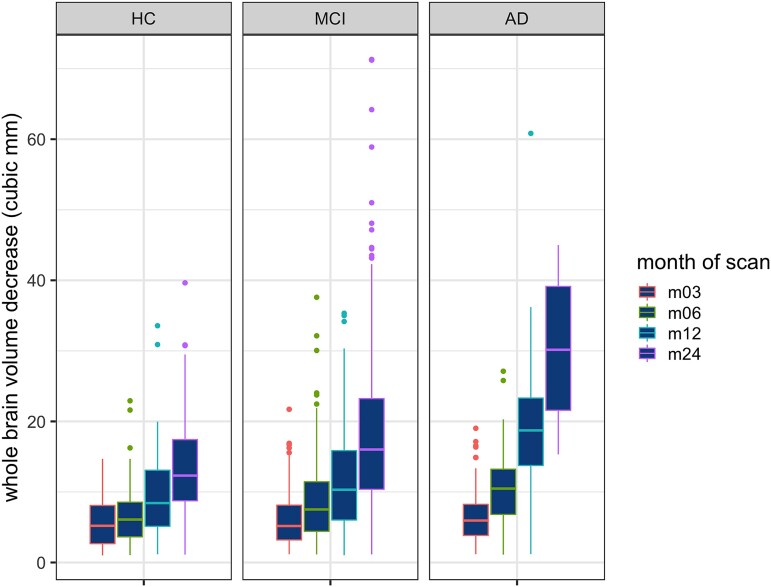
**Whole-brain atrophy (KN-BSI) in each diagnostic group across all time intervals.** m03, m06, m12 and m24 represent 3, 6, 12 and 24 months following baseline scan, respectively. Numbers of subjects in each group: HC (m03 = 92, m06 = 93, m12 = 105, m24 = 132); MCI (m03 = 171, m06 = 241, m12 = 268, m24 = 187); Alzheimer’s disease (m03 = 65, m06 = 67, m12 = 65, m24 = 15).

Table 1 shows the demographics of Core Data Sets 1 and 2, respectively. There were significant differences in age and years of education between the diagnostic categories. Supplementary Table 1 and Fig. 2 show the number of follow-up MRI scans available within each diagnostic group. The proportions match the sample size distributions. However, only a few scans were available at the 24-month mark for the Alzheimer’s disease subgroup.

**Table 1:** Baseline demographics of Core Data Set 1 and Core Data Set 2

	HC	MCI	Alzheimer’s disease	Total
*Core Data Set 1*				
*N*	218 (29.9%)	390 (53.4%)	122 (16.7%)	730
Female (%)	117(53.7)	171 (43.8)	51(41.8)	339 (46.4)
Age (SD)	73.4 (5.9)	71.5 (7.5)	74.8 (8.0)	72.6 (7.2)
Education (SD)	16.7 (2.5)	16.3 (2.6)	15.7 (2.7)	16.3 (2.6)
*Core Data Set 2*				
*N*	172 (28.6%)	336 (55.8%)	94 (15.6%)	602
Female (%)	91 (52.9)	151 (44.9)	37 (39.4)	279 (46.3)
Age (SD)	73.7 (6.1)	71.7 (7.3)	75.1 (7.5)	72.8 (7.1)
Education (SD)	16.6 (2.5)	16.4 (2.6)	15.8 (2.7)	16.3 (2.6)

For Core Data Set 1 more than half of the subjects fall in the MCI diagnostic category, almost a third HC and less than a fifth Alzheimer’s disease. The HC group has a higher fraction of females and as expected the Alzheimer’s disease participants are slightly older on average and there is a slight decrease in time spent in education for the Alzheimer’s disease subset (one-way ANOVA—age: *P* = 1.49e-05; education: *P* = 0.0064). In Core Data Set 2, we see a similar breakdown of sample sizes, sex, age and education by diagnostic group one-way ANOVA—age: *P* = 2.51e-05; education: *P* = 0.06). SD, standard deviation.

### White-matter lesions

WMH volume was greater in the Alzheimer’s disease group (Tukey multiple comparisons of means, Alzheimer’s disease-to-MCI:*p*_adj_ = 0.007, Alzheimer’s disease to HC:*p*_adj_ = 0.03) and marginally greater in the MCI group over the HC (Supplementary Fig. 1, a relationship, which holds when WMH is corrected for subject age; WMH volume was also greater in the Alzheimer’s disease group when broken down by sex; Supplementary Table 2). Following an exploration of regional WMH [including total frontal, parietal, temporal and occipital WMH volumes (results not shown)], the most compelling result was the combined basal ganglia and infratentorial WMH volumes in male subjects, which showed a clear difference between diagnostic groups (Supplementary Fig. 4).

### Polygenic risk

Our linear regression analysis for an effect between AD-PRS or CAD-PRS (over both PRS thresholds) on baseline grey matter (WBV), total WMH, or CSF amyloid did not find any statistically significant results (*P*_FDR_ > 0.05; Table 2). So as to explore whether AD-PRS and CAD-PRS effects are independent, we have rerun the model where both AD-PRS and CAD-PRS are predictive variables for WMH and WBV (corresponding parameters in Supplementary Table 3). This shows that in the oligogenic regime, both AD-PRS and CAD-PRS are statistically associated with WBV in the MCI group. Also in this regime CAD-PRS and WBV are associated with HC. Finally, AD-PRS is correlated with WMH in the Alzheimer’s disease group. This shows that in the case of AD-PRS with CAD-PRS as a covariate and CAD-PRS with AD-PRS as a covariate, certain associations are strengthened. In the MCI group, they seem to be driving one another, but there are also examples of independent association. Of note, there is no correlation between AD-PRS and CAD-PRS in the oligogenic regime (Pearson correlation coefficient = 0); however, in the polygenic regime, there is a small anticorrelation (Pearson correlation coefficient = −0.33). However, for individuals with an Alzheimer’s disease diagnosis, both AD-PRS (*P*_FDR_ = 0.03) and CAD-PRS (*P*_FDR_ = 0.04) are associated with occipital lobe WMH at the PRS threshold of *P* = 0.5 (Table 3).

**Table 2:** Results from Core Data Set 1 using the low *P*-cut-off 1e-05 and the high *P*-cut-off 0.5

		HC		MCI		Alzheimer’s disease	
		AD-PRS	CAD-PRS	AD-PRS	CAD-PRS	AD-PRS	CAD-PRS
*P* = 1e-05	WBV	0.61 (0.30)	1.9 (0.27)	−2.2 (0.07)	1.8 (0.34)	1.0 (0.49)	−0.78 (0.47)
	WMH (total)	0.21 (0.44)	0.31 (0.39)	−1.08 (0.34)	−1.43 (0.48)	1.93 (0.09)	−0.78 (0.13)
	Amyloid Beta	−0.89 (0.20)	2.4 (0.16)	0.1 (0.48)	1.1 (0.47)	−0.35 (0.38)	0.13 (0.45)
*P* = 0.5	WBV	−0.8 (0.30)	0.85 (0.26)	−0.96 (0.31)	0.24 (0.42)	0.31 (0.49)	−0.23 (0.47)
	WMH (total)	0.68 (0.36)	0.48 (0.39)	−0.73 (0.34)	−0.12 (0.48)	1.63 (0.09)	1.35 (0.13)
	Amyloid Beta	−0.89 (0.20)	0.13 (0.48)	−0.46 (0.48)	−0.49 (0.47)	−1.2 (0.20)	1.8 (0.12)

*t*-values (*P*-values, one-tailed false discovery rate-corrected) to 2 decimal points following linear regression with confounders for age, sex, education, *APOE*-e4 burden and first five principal components of population structure (additional ICV confounder for log-transformed WMH regression) across diagnostic groups for AD-PRS and coronary artery disease-PRS; WBV normalized by ICV; AD-PRS and coronary artery disease-PRS both exclude *APOE* region. For amyloid beta, subjects with missing data were removed from the linear model (*n* = 665).

**Table 3:** Regional WMH analysis from Core Data Set 1 using the low *P*-cut-off 1e-05 and the high *P*-cut-off 0.5

		HC		MCI		Alzheimer’s disease	
		AD-PRS	CAD-PRS	AD-PRS	CAD-PRS	AD-PRS	CAD-PRS
*P* = 1e-05	Frontal	0.29 (0.45)	0.24 (0.41)	−0.98 (0.34)	−2.0 (0.21)	1.8 (0.29)	−1.2 (0.25)
	Parietal	−0.15 (0.49)	0.52 (0.45)	−1.0 (0.49)	−0.95 (0.49)	1.8 (0.12)	−1.5 (0.16)
	Occipital	0.66 (0.45)	0.29 (0.46)	−1.1 (0.17)	−1.6 (0.5)	1.4 (0.06)	−0.96 (0.2)
	Temporal	−0.03 (0.44)	1.2 (0.31)	−0.13 (0.42)	−1.3 (0.45)	2.3 (0.38)	−0.69 (0.28)
	Basal ganglia + thalami + infratentorial	0.28 (0.46)	−0.15 (0.47)	−1.0 (0.11)	−0.53 (0.46)	0.93 (0.49)	−0.21 (0.47)
*P* = 0.5	Frontal	0.64 (0.38)	0.76 (0.27)	−0.26 (0.34)	−0.10 (0.49)	1.1 (0.29)	0.80 (0.25)
	Parietal	0.52 (0.49)	0.29 (0.45)	−0.47 (0.47)	−0.09 (0.49)	1.5 (0.12)	1.2 (0.16)
	Occipital	0.48 (0.34)	0.27 (0.46)	−0.92 (0.17)	0.03 (0.5)	**1.7** (**0.03)**	**2.0** (**0.04)**
	Temporal	0.53 (0.34)	0.69 (0.31)	−1.3 (0.20)	−0.31 (0.45)	2.0 (0.39)	1.3 (0.17)
	Basal ganglia + thalami + infratentorial	0.71 (0.46)	−0.42 (0.45)	**−0.71** (**0.05)**	0.15 (0.46)	0.76 (0.49)	0.35 (0.47)

*t*-values (*P*-values, one-tailed false discovery rate-corrected) to 2 deimal points following linear regression with confounders for age, sex, education, *APOE*-e4 burden and first five principal components of population structure and natural log of regional WMH normalized by regional volume. Statistically significant results are given in bold font.

### Whole-brain atrophy

Given that a snapshot of WBV at baseline may not be sufficiently informative in that it represents just one instance along the disease-course trajectory, we investigated the effect of total WMH at baseline and PRS on longitudinal changes in WBVs. Following the baseline scan, KN-BSI increases (representing a decrease in WBV) with passing months in all three diagnostic groups (Fig. 2), but this is most pronounced in the Alzheimer’s disease cohort (see Supplementary Fig. 4 for multiple comparison *P*-values). Using linear regression, we found a statistically significant effect (*P*_FDR_ < 0.05) of baseline WMH on WBV atrophy in all diagnostic groups over differing times (Table 4). This association also held in a corresponding regional analysis of frontal, parietal, occipital, temporal and combined basal ganglia, thalami and infratentorial WMH volumes (Supplementary Table 4), with WBV drop being particularly correlated over Months 12 and 24 (especially in the MCI cohort).

**Table 4:** Results from Core Data Set 2

		HC			MCI			Alzheimer’s disease		
KN-BSI	PRS cut-off	WMH	AD-PRS	CAD-PRS	WMH	AD-PRS	CAD-PRS	WMH	AD-PRS	CAD-PRS
Month 3	*P* = 1e-5	1.9 (0.053)	0.89 (0.48)	0.68 (0.49)	1.5 (0.14)	**2.7** (**0.01)**	0.17 (0.46)	**2.9** (**0.0039)**	0.84 (0.48)	1.2 (0.27)
	*P* = 0.5		−0.18 (0.47)	−0.04 (0.49)		−0.36 (0.4)	1.9 (0.07)		−0.18 (0.48)	0.95 (0.27)
Month 6	*P* = 1e-5	1.9 (0.062)	1.8 (0.2)	1.1 (0.3)	**2.3** (**0.019)**	**3.1** (**0.004)**	−0.004 (0.5)	**2.49** (**0.013)**	1.5 (0.32)	0.58 (0.3)
	*P* = 0.5		0.57(0.39)	0.6(0.3)		−0.19 (0.49)	1.8 (0.08)		0.2 (0.49)	1.7 (0.1)
Month 12	*P* = 1e-5	**4.2 (3.7e-05)**	**2.6** (**0.04)**	0.64 (0.49)	**2.5** (**0.013)**	**2.8** (**0.01)**	−0.007 (0.5)	**3** (**0.0024)**	1.6 (0.27)	−0.23 (0.46)
	*P* = 0.5		1.7 (0.06)	−0.03 (0.49)		−0.49 (0.35)	**2.2** (**0.03)**		−0.77 (0.28)	1.5 (0.15)
Month 24	*P* = 1e-5	**2.7 (0.0076)**	**3.3** (**0.007)**	0.63 (0.45)	1.3 (0.2)	**3.1** (**0.004)**	−0.28 (0.4)	0.19 (0.85)	2.3 (0.14)	−0.25 (0.4)
	*P* = 0.5		**2.5** (**0.01)**	−0.26 (0.45)		−0.5 (0.37)	**2.1** (**0.04)**		−0.77 (0.3)	0.6 (0.36)

*t*-values (*P*-values) for a range of KN-BSI whole-brain atrophy over scans at Months 3, 6, 12, and 24 with respect to natural log of WMH (at baseline or Month 0), CAD-PRS and AD-PRS in different diagnostic groups; linear regression with confounders for age, sex, education, *APOE*-e4 burden and five principal components of population structure (including ICV confounder for WMH regression) across diagnostic groups for two PRS thresholds; *APOE* region excluded in AD-PRS and CAD-PRS. All PRS regression *P*-values are corrected for multiple testing and are one-tailed. Statistically significant results are given in bold font.

AD-PRS is associated with whole-brain atrophy (KN-BSI) in HC and MCI, but not in subjects with Alzheimer’s disease. Moreover, in the MCI cohort AD-PRS is correlated with brain atrophy at each time point but only for the *P* = 1e-5 PRS threshold. Finally, in the HC cohort, the KN-BSI and AD-PRS are correlated in the later Months 12 and 24.

There is no statistically significant correlation between CAD-PRS and KN-BSI in both the HC and Alzheimer’s disease diagnostic groups. However, in the MCI cohort, we see a correlation in the later Months 12 and 24, but only for the *P* = 0.5 threshold. So in the *P* = 0.5 threshold for Months 12 and 24, the CAD-PRS is more correlated with whole-brain atrophy than the AD-PRS (Month 12: *t* = 2.2, *P*_FDR_ = 0.03; Month 24: *t* = 2.1, *P*_FDR_ = 0.04).

### Whole-brain atrophy acceleration

**Figure 3:**
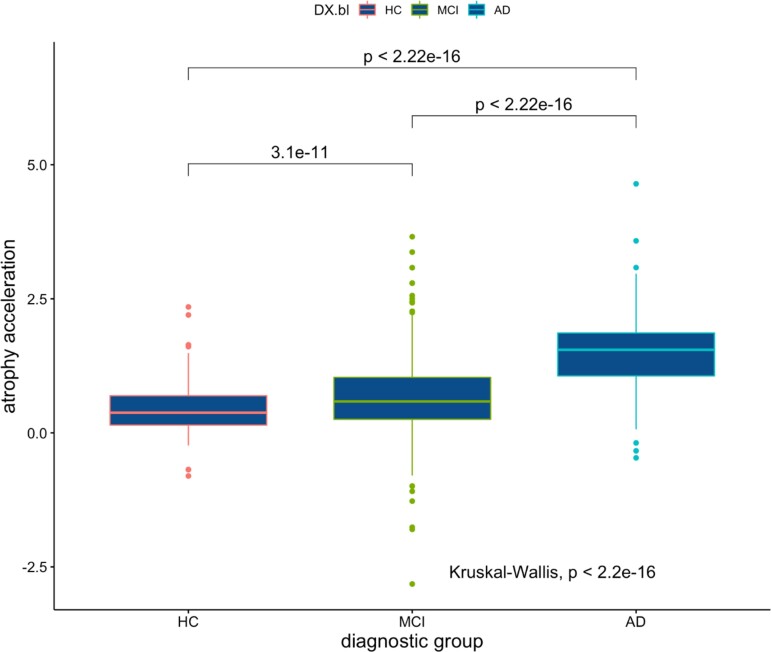
**Median atrophy acceleration in cm^3^/month^2^ across diagnostic groups.** Alzheimer’s disease subjects WBV is decreasing much faster than MCI, which are decreasing faster than HC. Multiple comparison Kruskal–Wallis *P*-values shown. Kruskal–Wallis chi-squared = 256.55, d.f. = 2, *P*-value < 2.2e-16.

To probe the *rate of change* of WBV over longitudinal scans per subject with respect to the underlying genetics, we enumerate the ‘atrophy acceleration’ in Table 5, top. *Atrophy acceleration* is significantly greater in the Alzheimer’s disease cohort compared with HC and MCI (*P* < 2.2e-16; Fig. 3). While there is no correlation between CAD-PRS and atrophy acceleration (Table 5, top), there is a statistically significant correlation between AD-PRS and atrophy acceleration for the *P* = 0.5 threshold in the HC. It is also notable that at the *P* = 1e-5 threshold, this correlation shows a statistical trend (*P*_FDR_ = 0.06). Our mixed effect analysis (Table 5, bottom) showed that there is no association between longitudinal changes in grey matter (quantified by the BSI) and time-by-CAD polygenic score interaction; however, time-by-Alzheimer’s disease-polygenic score interaction is correlated with longitudinal WBV changes in both the HC (*P*_FDR_ = 0.01) and MCI (*P*_FDR_ = 0.03) subsets at the *P* = 1e-5 PRS threshold.

**Table 5:** Association between atrophy acceleration and polygenic risk. Top: Atrophy acceleration, or rate of change of WBV (gradient from line of best fit over serial scans at months 3, 6, 12 and 24 per subject) with respect to AD-PRS and CAD-PRS. All PRS regression *P*-values are corrected for multiple testing and are one-tailed. Bottom: Atrophy acceleration estimated from time-by-PRS interaction in linear mixed effect models

Diagnostic group	PRS cut-off	CAD-PRS *t*-value	CAD-PRS *P*-value	AD-PRS *t*-value	AD-PRS *P*-value
**Gradient based**					
Healthy control	*P* = 1e-5	−1.35	0.28	1.84	0.06
	*P* = 0.5	0.58	0.35	**2.06**	**0.04**
Mild cognitive impairment	*P* = 1e-5	−1.98	0.26	0.77	0.49
	*P* = 0.5	−0.49	0.34	−0.16	0.49
Alzheimer’s disease	*P* = 1e-5	0.35	0.47	−1.21	0.49
	*P* = 0.5	−0.3	0.47	0.03	0.49
**Mixed Effect Model**					
					
Healthy control	*P* = 1e-5		0.46		**0.01**
	*P* = 0.5		0.51		**0.05**
Mild cognitive impairment	*P* = 1e-5		0.27		**0.03**
	*P* = 0.5		0.58		0.16
Alzheimer’s disease	*P* = 1e-5		0.31		0.97
	*P* = 0.5		0.86		0.72

Statistically significant results are given in bold font.

## Discussion

In this study, we aimed to investigate possible associations between cardiac genetics and Alzheimer’s dementia. We focused on the genetic variants outside of the *APOE* locus that have been shown to be associated with Alzheimer’s disease and, separately, with CAD genetic variants. In particular, we looked at their impacts on white-matter lesions and WBV changes. We found a correlation between coronary artery genetic risk and whole-brain atrophy suggesting that many small-effect-size variants contribute to neuronal loss in MCI individuals. Surprisingly, under certain PRS thresholds and at certain times in the disease course, the underlying polymorphisms for CAD may well have more of an impact than those of Alzheimer’s disease. We have also highlighted how whole-brain atrophy acceleration is associated with AD-PRS outside the *APOE* locus in healthy individuals. Further, we have shown for the first time that genetic variants that contribute to both Alzheimer’s disease and CAD beyond *APOE* are a strong predictor of white-matter lesions in the occipital lobe in subjects already diagnosed with Alzheimer’s disease.

White-matter lesions were clearly higher in the Alzheimer’s disease group, something that is driven in part by age (Supplementary Fig. 5). While all linear models treated age as a covariate, disentangling white-matter lesion increase as a function of time separate to any age-related Alzheimer’s disease pathologies is challenging. This may well be of great importance if early pharmaceutical intervention is necessary to curtail Alzheimer’s disease in later life, as WMH extent could be utilized as a surrogate biomarker for downstream dementia.^43^

No associations were found between AD-PRS or CAD-PRS and baseline total WBV and WMH volumes across all diagnostic groups, although PRSs have been shown to be predictive of Alzheimer’s disease risk.^44^ However, the analysis of AD-PRS with CAD-PRS as a covariate and CAD-PRS with AD-PRS as a covariate showed that certain associations are strengthened. In particular, in the MCI group the PRSs seem to be driving one another but there are also examples where the PRSs are independent; in most cases, there are no new associations. That said, AD-PRS beyond *APOE* is correlated with white-matter load in the occipital lobe in Alzheimer’s disease subjects upon the inclusion of many variants (polygenic *P* = 0.5). In fact, our regional analysis of WMH showed a statistically significant association between both AD-PRS and CAD-PRS with occipital lobe WMH lesion volume in the Alzheimer’s disease group at a PRS threshold of *P* = 0.5. This confirms the presence of significant WMH lesions in this lobe seen in other dementia cohorts^45^ and the finding that occipital WMH is correlated with reduced executive function.^46^ This may also be related to the cerebral amyloid angiopathy pathway. We were unable to establish this white-matter load association by the occipital lobe layer; however, this is, to our knowledge, the first confirmation of a genetic link with this regional WMH and a genetic link to CAD.

There are also no statistically significant correlations with CSF amyloid beta and CAD-PRS or AD-PRS; presumably, we did not find evidence for an association with AD-PRS because amyloid biomarkers are mainly influenced by the *APOE*-e4 genotype.^25^ Moreover, the analysis was conducted within disease groups and not between disease groups, where stronger differences are known to exist.^47^

When it came to investigating whole-brain changes over time, our analysis confirmed an association between baseline WMH and WBV decline across all three diagnostic groups.

WMH here was derived from scans at baseline and not at the time of repeat scans, thus, WMH burden may indeed serve as an indicator of near-term or future decreases in WBV. The strength of these associations was driven by sample size and time between baseline and follow-up WBV measure. These results confirm earlier studies^45,48^ that showed higher white-matter lesion load is correlated with decreasing WBV, albeit using cross-sectional WBV measures. Earlier studies investigating longitudinal WBV change only found an association with WMH in HC^49^ or hippocampal atrophy in MCI subjects,^50^ in contrast to our analysis which extends this correlation to participants with Alzheimer’s disease.

In the matter of whether PRS are predictive of longitudinal decline in WBV, our analysis showed correlations between AD-PRS and WBV decline in the HC group (later months) and MCI group (all months), but not the Alzheimer’s disease group. The decline in the HC group reflects regional WBV decline with respect to AD-PRS observed previously.^51^ The lack of a polygenic effect in the Alzheimer’s disease group, may originate from either limited statistical power (a power analysis using the ‘pwr’ R library shows a correlation coefficient of *r* = 0.16 can be detected at 80% power in the Alzheimer’s disease sample), the overall extent of WBV damage in Alzheimer’s disease or that while Alzheimer’s disease is advanced, WBV volume decrease is driven by other factors beyond Alzheimer’s disease–risk variants. As in the HC group, the AD-PRS, like baseline WMH, is correlated with WBV decrease in later Months 12 and 24, which raises the question as to whether genetic effects become more influential with time. Before this can be answered, we would have to account for there being more samples in Months 12 and 24 and the fact that picking up changes in WBV volumes over Months 3 and 6 will be more difficult as they will likely be more subtle. Interestingly, the AD-PRS correlation with WBV atrophy in the MCI group was significant at all time points for the oligogenic (*P* = 1e-5) PRS threshold but not the polygenic (*P* = 0.5) PRS threshold. This suggests that there are some larger effect sizes, common variants (i.e. the peaks in the Manhattan plot outside the *APOE* locus) that are predictive of brain atrophy in persons that already display some cognitive impairment. This result supports the hypothesis that a small number of Alzheimer’s disease-SNPs allow maximal predictive power in Alzheimer’s disease–related subjects.^52^ MCI, however, is a heterogeneous group of which only a subset exhibit MCI due to an underlying Alzheimer’s disease pathology. This is also the most bimodal group in terms of whether they have Alzheimer’s disease (progressors vs. not progressors). Thus, the AD-PRS may correctly predict WBV decline in subjects who have MCI due to Alzheimer’s disease.

The motivation for this study was an exploration of the genetic effects of CAD on Alzheimer’s disease. The CAD-PRS was only correlated with WBV decline in the MCI group reflecting incidence-based relationships seen in other studies.^53^ Again, the PRS-WBV atrophy association was strongest in the later Months 12 and 24 but showed a statistical trend in the earlier Months 3 and 6. In the MCI group, CAD-PRS is significantly correlated with WBV atrophy for polygenic effects (*P* = 0.5 thresholds) but AD-PRS is significantly correlated with WBV atrophy for oligogenic effects (*P* = 1e-5 threshold). One interpretation might be that the more genetic variants included, the more important the role of cardiac genetics over Alzheimer’s disease genetics in individuals with MCI. Another interpretation may be that AD-PRS is contributing to individuals with MCI due to developing Alzheimer’s disease dementia, whereas CAD-PRS is bestowing subjects with a more vascular component to their cognitive loss. It is also important to highlight here that while we investigated the CAD-PRS effects, we did not investigate the contribution of cardiometabolic health itself.

Recent work^54^ investigating the genetic architecture of Alzheimer’s disease with regards to PRS thresholds argues that the polygenic threshold is optimal. Their work shows that studies using an oligogenic threshold ignore the fact that there will be fewer *APOE*-e4 carrying individuals in the older category, biasing results against high *P*-value threshold variants. In our study, we removed the *APOE* locus so as to explore genetic changes outside this genetic region; however, all linear regressions include *APOE*-e4 burden as a confounding variable. Investigators have shown that *APOE*’s effect on Alzheimer’s disease is greater in older cohorts and suggest that variants outside of *APOE* could contribute to Alzheimer’s disease in older persons.^55^ As *APOE* has been considered a target for both the treatment of coronary heart disease and Alzheimer’s disease, these two pathologies may both be affected by similar pathways. We examined regression models with and without the *APOE*-e4 covariate using ANOVA. The inclusion of the *APOE*-e4 burden improved all models significantly (*p*_adj_ < 0.01) with the exception of WBV volume decline in Months 3 (*p*_adj_ = 0.8) and 6 (*p*_adj_ = 0.1) in the HC group (results not shown).

Brain volume is decreasing faster in Alzheimer’s disease subjects than in MCI subjects, where it is decreasing faster than in healthy individuals (Fig. 3). The study of polygenic risk suggests that many small effect size Alzheimer’s disease variants beyond *APOE* are a predictor of atrophy acceleration in healthy individuals (and perhaps also MCI subjects). This is in many ways a surprising result as one would expect the Alzheimer’s disease cohort (and perhaps the MCI cohort) to be comprised of persons whose WBV decrease is accelerating as the pathology develops. Some caution is warranted here given that Alzheimer’s disease cohort subjects have fewer longitudinal scans, thereby biasing the computed gradient. That said, the HC correlation may indicate that genetic effects modulate the speed at which brain cells atrophy during healthy stages. What we do not know is whether a larger atrophy acceleration while healthy makes Alzheimer’s disease inevitable, more likely or in no way indicative of downstream pathology. However, it is reassuring that the AD-PRS in HC is corroborated in the mixed-effects models, which are more flexible, embodying both random intercepts and random slopes so as to more realistically capture the subject-level heterogeneity.

The BSI data available through ADNI are not mid-point symmetric: WBV change going from Scan A-to-C is not the same as adding WBV volume changes in Scan A-to-B and Scan B-to-C. The implication is that one has to be cautious about saying anything about the time-varying nature of these non-symmetric BSIs. We investigated this effect using symmetric BSI data^56^ for the same ADNIGO and ADNI2 subjects (*n* = 572; 3T, accelerated, identical scanner protocol, removing BSI < 0, only Months 3, 6, 12 and 24 post-baseline). Repeated analysis of this symmetric BSI data resulted in the same statistically significant outcomes as described in this work (results not shown). The only exception to this is that the correlation between baseline WMH and WBV atrophy in the HC group was no longer replicated. We are also cautious about overinterpreting our results: while the BSI does measure changes at the border of the brain, it does not mean that the change has actually occurred there. Some tissue may have been lost from the middle of the white matter, with this change being measured at the edge of the brain.

Given that both whole-brain atrophy and whole-brain atrophy acceleration are both shown to be correlated with polygenic scores, it may be that utilizing such genetic summary information (perhaps alongside other routinely collected health measures) can 1 day be used at birth (or middle age) as a predictor of late-life cognitive problems.^57^ If so, and if such risk is driven in part by cardiac health, it may be that this risk can be reduced through wellness and behaviour changes in, early to mid-life.^58^ Of course, this does depend on whether healthy individuals with such brain changes go on to develop dementia, something only larger longitudinal studies over many years can answer.

## Conclusions

The link between CAD and Alzheimer’s disease is gaining more attention, with one recent study identifying 23 brain regions associated with both cardiovascular disease and Alzheimer’s dementia.^59^ WMH loads have been linked to hypertension, hypercholesterolaemia and body mass index in middle-aged subjects, all of which also contribute to cardiac health.^60^ While our work offers some support for the importance of genetic variants known to affect coronary heart disease also being involved in cognitive decline in the elderly, more data are needed to validate these findings. Similarly, the usefulness of WMHs and atrophy accelerations needs to be investigated further. In particular, serial WMH measures alongside WBV changes would be a more realistic guide to cerebral changes. Further understanding of this pathway from gene to brain will come from cardiac imaging of the heart linked to CAD genetics alongside routinely measured biomarkers over the life-course of many individuals, such as those found in large-scale, data-rich, longitudinal cohort studies.

## Supplementary Material

fcac314_Supplementary_Data

## Data Availability

ADNI data (WBV’s and BSI’s) are available through an application on the ADNI website. Derived data used in this study (PRS’s and WMHs) are available from the authors upon request.

## Abbreviations

ADGC =: Alzheimer’s Disease Genetics Consortium
ADNI =: Alzheimer’s disease neuroimaging initiative
*APOE* =: apolipoprotein
BaMoS =: Bayesian model selection
BSI =: boundary shift integral
CAD =: coronary artery disease
FDR =: false discovery rate
GWAS =: genome-wide association studies
HC =: healthy control
ICV =: intracranial volume
LD =: linkage disequilibrium
MCI =: mild cognitive impairment
MAF =: minor allele frequency
PRS =: polygenic risk score
SNP =: single-nucleotide polymorphism
WBV =: whole-brain volume
WMH =: white-matter hyperintensity
