# Supplementary material for: Polygenic coronary artery disease association with brain atrophy in the cognitively impaired
Source: Brain Commun. 2022 Nov 30;4(6):fcac314. doi: 10.1093/braincomms/fcac314 (PMC9746681; doi:10.1093/braincomms/fcac314)
Supplement: fcac314_Supplementary_Data [file fcac314_supplementary_data.pdf]

Supplementary material

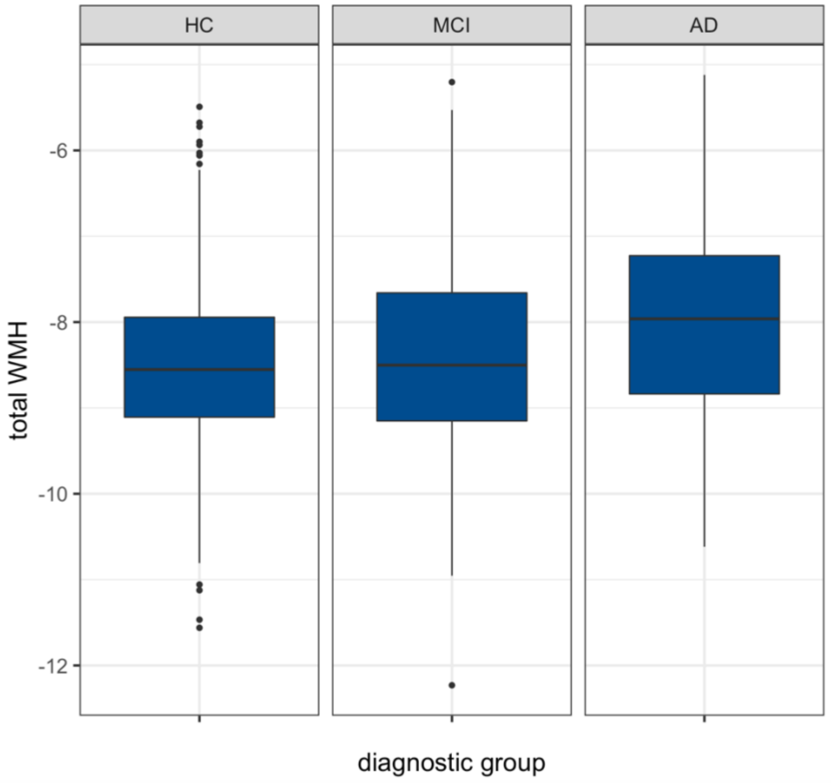

Supplementary Figure 1: Boxplots of natural-log transformed total WMH normalised by ICV for all subjects across diagnostic groups, healthy control (HC), mild cognitive impairment (MCI) and Alzheimer's disease (AD). HC (N=218), MCI (N=390), AD (N=122). MCI-HC (Tukey HSD test; diff=-1.7e-8, lwr=-1.6e-7, upr=1.2e-7, adjusted p=0.96), AD-HC (Tukey HSD test; diff=2.04e-7, lwr=1.8e-8, upr=3.9e-7, adjusted p=0.028), AD-MCI (Tukey HSD test; diff=2.2e-7, lwr=4.9e-8, upr=3.9e-7, adjusted p=0.0070).

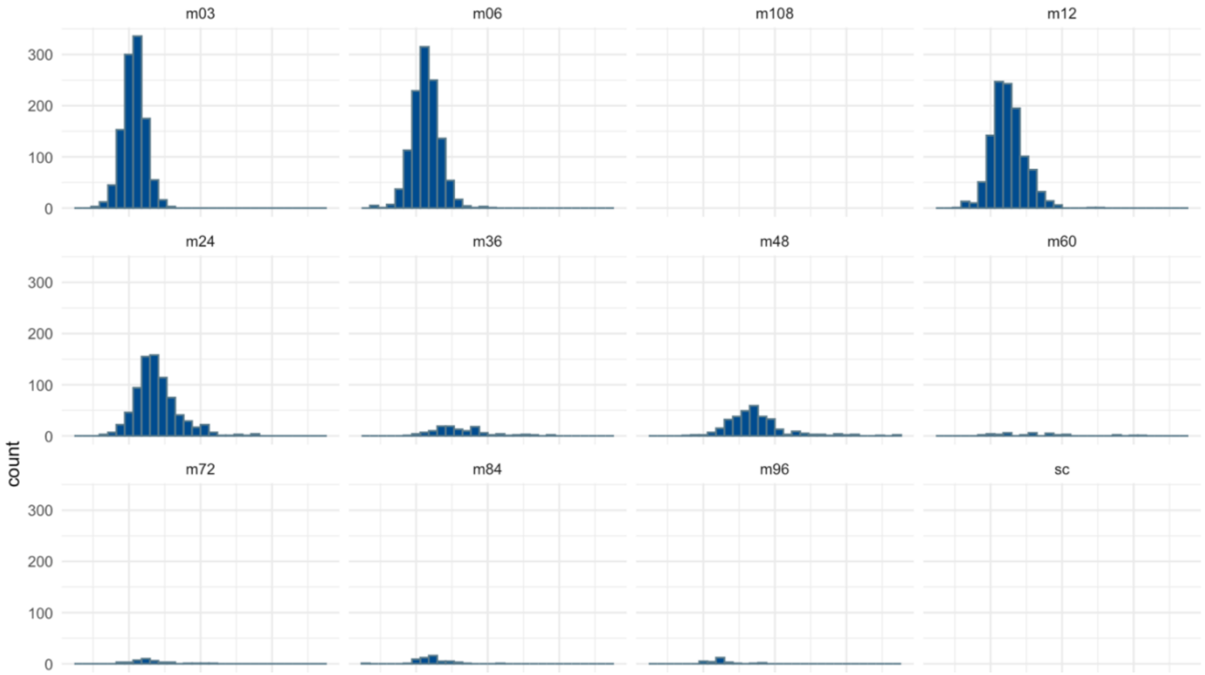

Supplementary Figure 2: Number of longitudinal BSI scans in CORE DATASET 1. Number of scans: m03=1247, m06=1330, m12=1318, m24=1050, m36=202, m48=513, m60=117, m72=163, m84=99, m96=44

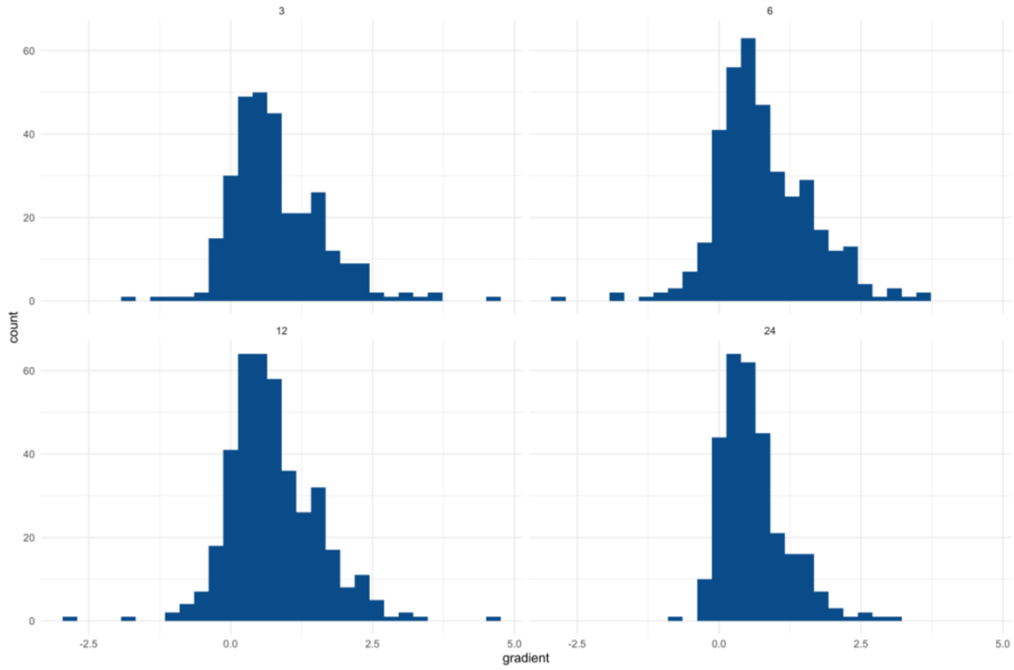

Supplementary Figure 3: Number of longitudinal BSI in CORE DATASET 2 over months 3, 6, 12 and 24. Number of scans: month3 = 328, month6 = 401, month12 = 438, month24 = 334

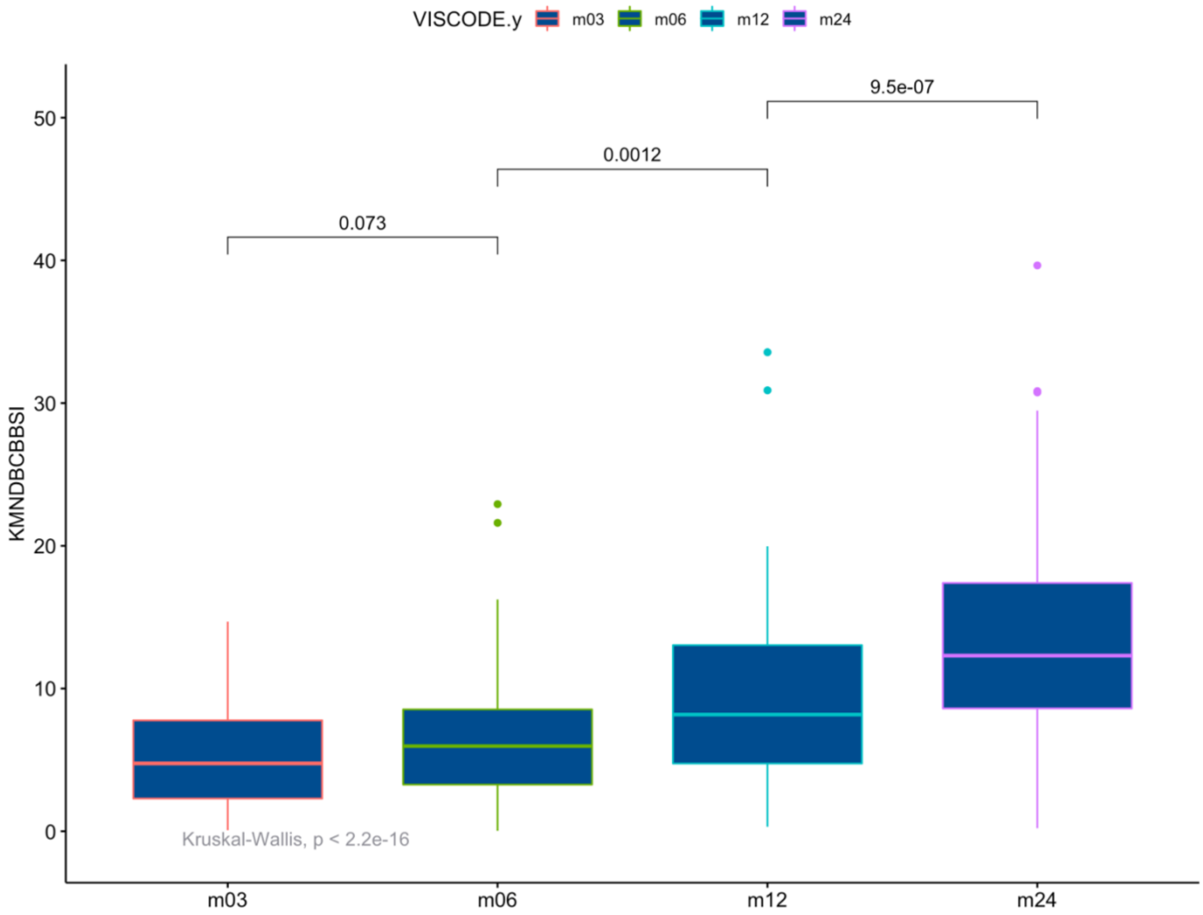
VISCODE.y
m03
m06
m12
m24
0.073
0.0012
9.5e-07
KMNDBCBBSI
50
40
30
20
10
0
Kruskal-Wallis, p < 2.2e-16
m03
m06
m12
m24

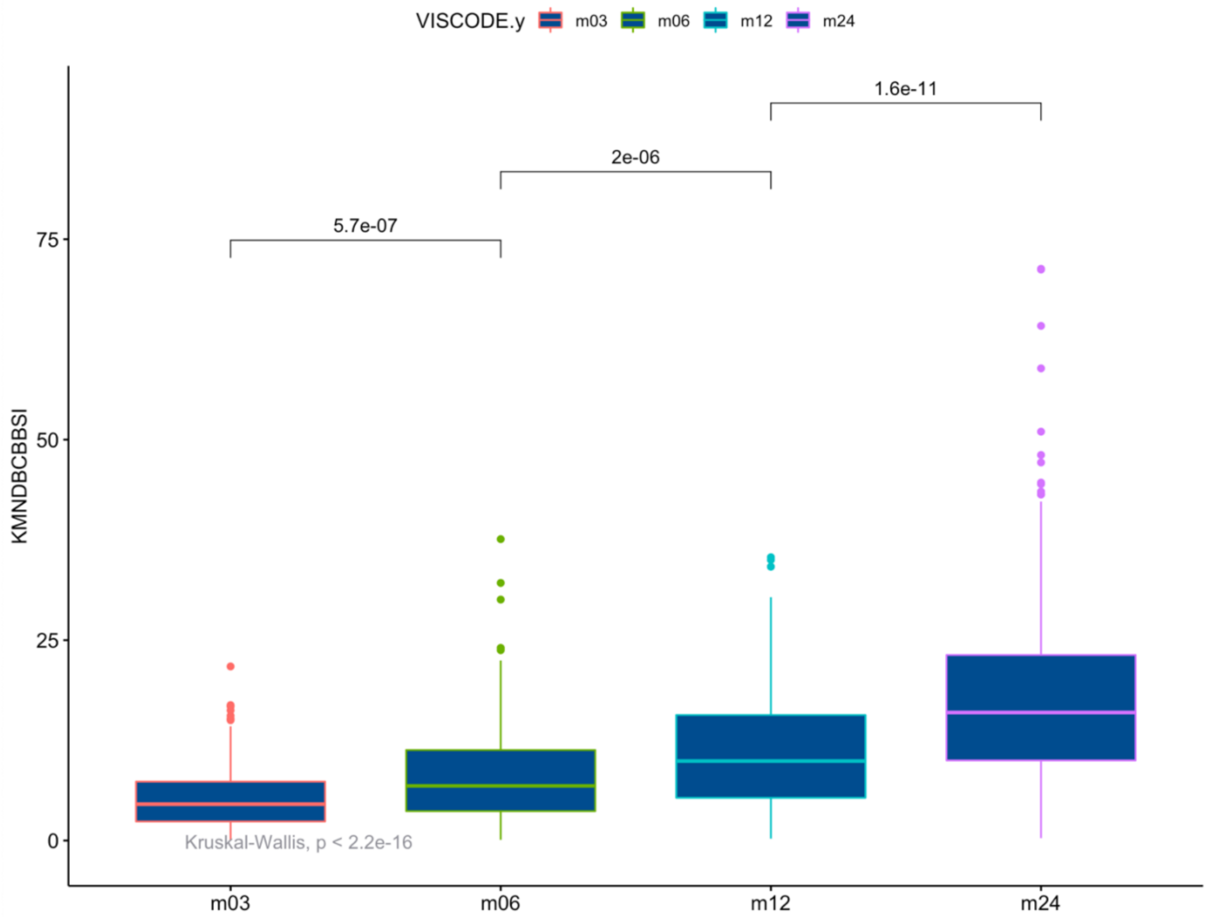

VISCODE.y
m03
m06
m12
m24
1.6e-11
2e-06
5.7e-07
75
50
25
0
KMNDBCBBSI
Kruskal-Wallis, p < 2.2e-16
m03
m06
m12
m24

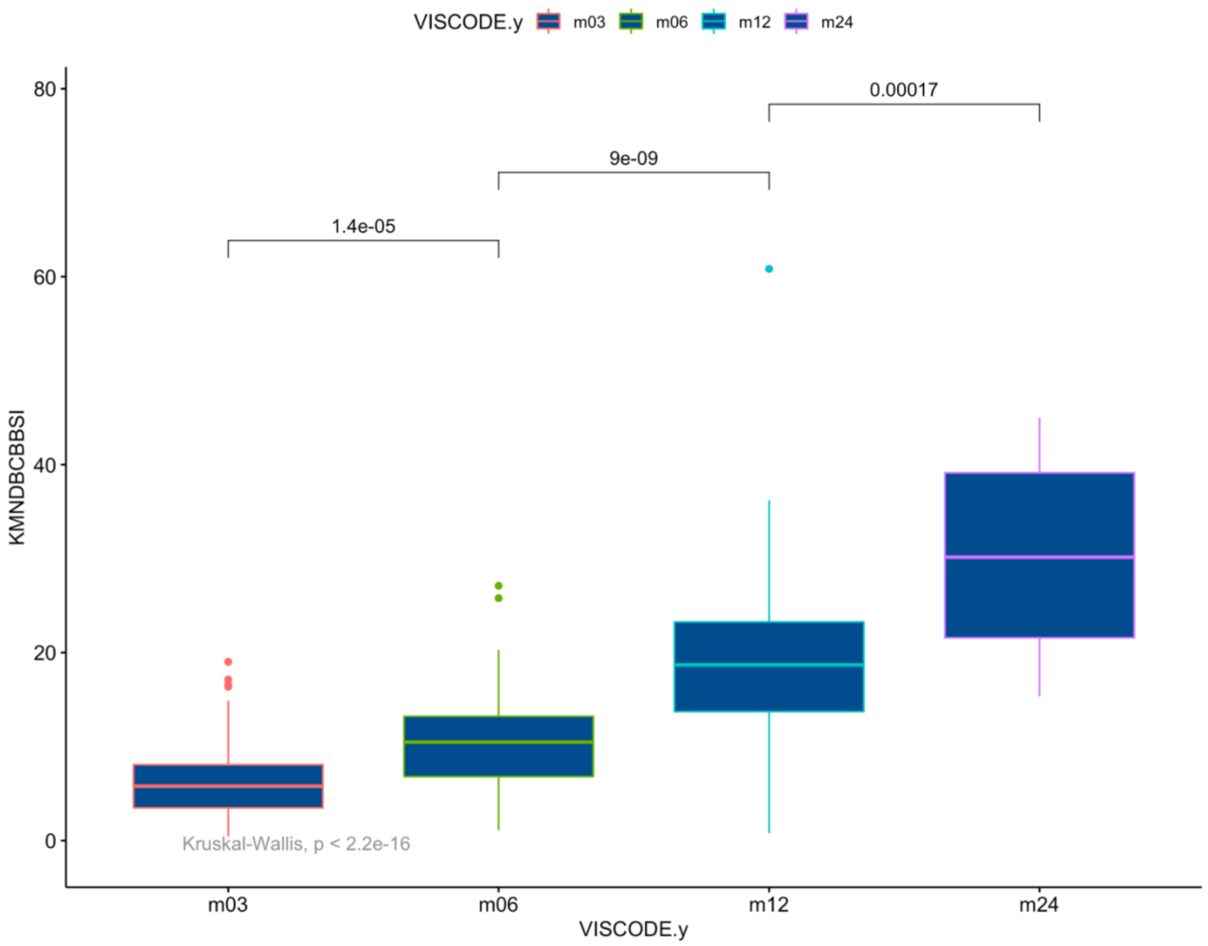

Supplementary Figure 4: Kruskal-Wallis multiple pairwise comparisons between scan months over diagnostic groups HC=422, Kruskal-Wallis chi-squared = 105.97, df = 3, p-value < 2.2e-16 (top), MCI=867, Kruskal-Wallis chi-squared = 198.88, df = 3, p-value < 2.2e-16 (middle) and AD=212, Kruskal-Wallis chi-squared = 95.569, df = 3, p-value < 2.2e-16 (bottom). Multiple comparisons pairwise p-values shown on plot.

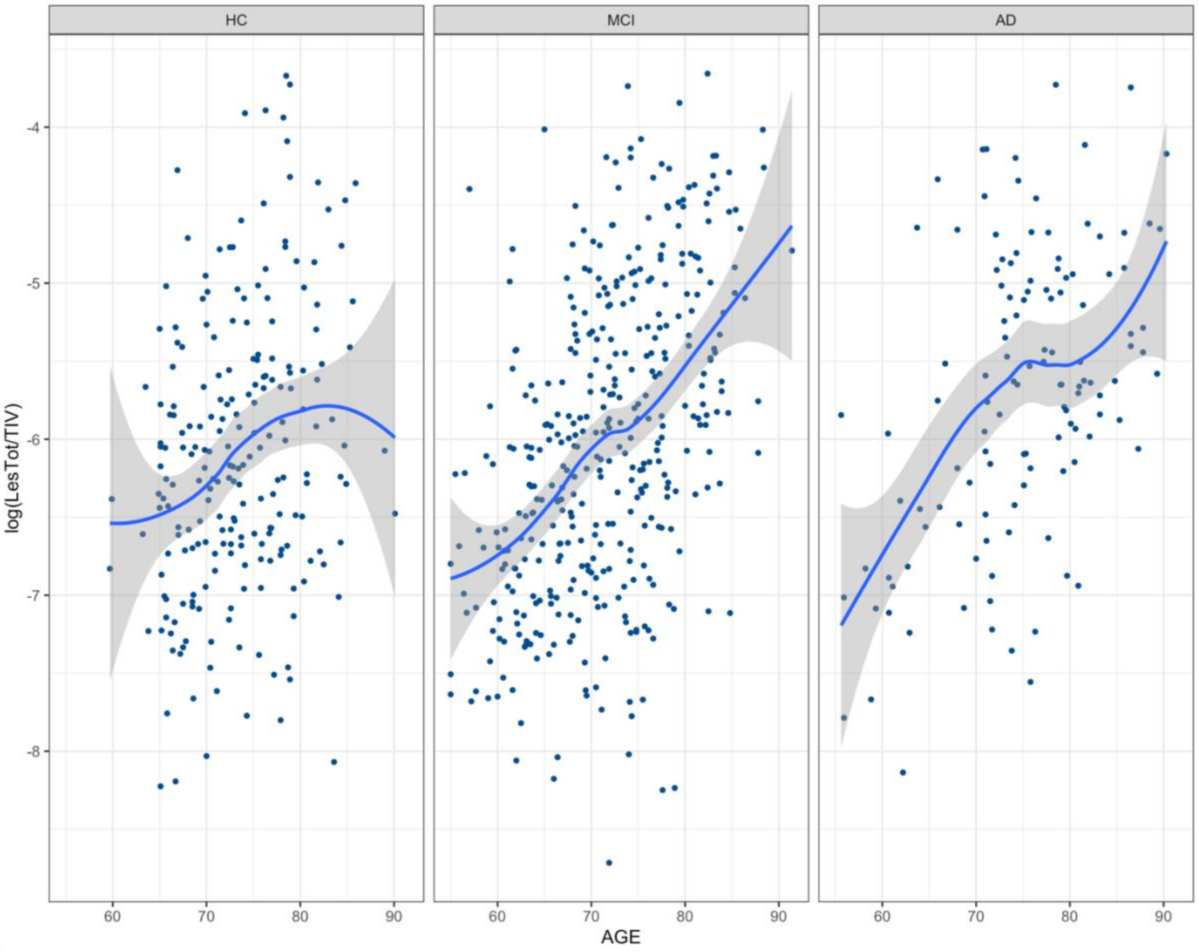

Supplementary Figure 5: Correlation between age and WMH load. Natural log transformed normalised (for total intercranial volume) total WMH versus age (years) across diagnostic groups for CORE DATASET 1. Overlayed local polynomial regression fit with smoothing and Pearson's product-moment correlation (HC: n=218, t=3.9, p=0.0001; MCI: n=390, t=10.7, p<2.2e-16; AD: n=122, t=5.9, p=2.3e-8).

Supplementary Table 1: Numbers of scans per diagnostic group per time scan was take for CORE DATASET 2

	HC	MCI	AD	total
month 3	92	171	65	328
month 6	93	241	67	401
month 12	105	268	65	438
month 24	132	187	15	334
total	422	867	212	1501

Supplementary Table 2: Tukey multiple comparisons of means for natural-log transformed total WMH normalised by ICV stratified by sex across diagnostic groups, healthy control (HC), mild cognitive impairment (MCI) and Alzheimer's disease (AD)

	female (adjusted p-value)	male (adjusted p-value)
**MCI-HC**	0.99	0.41
**AD-HC**	0.05	0.004
**AD-MCI**	0.03	0.04

Supplementary Table 3: Results from CORE DATASET 1 using the low p-cut-off 1e-05 and the high p-cut-off 0.5 with both AD-PRS and CAD-PRS as predictive variables. t-values (p-values, one-tailed FDR-corrected) to 2sf. following linear regression with confounders for age, sex, education, *APOE*-e4 burden and first five principal components of population structure (additional ICV confounder for log transformed WMH regression) across diagnostic groups for AD PRS (with CAD-PRS covariate) and CAD PRS (with AD-PRS covariate); WBV normalised by ICV; AD PRS and CAD PRS both exclude *APOE* region.

		HC		MCI		AD	
		AD PRS	CAD PRS	AD PRS	CAD PRS	AD PRS	CAD PRS
P=1e-05	**WBV**	0.57 (0.29)	1.9 (0.03)	-2.2 (0.01)	1.91 (0.03)	1.0 (0.15)	-0.79 (0.22)
	**WMH (tot)**	0.21 (0.42)	0.30 (0.38)	-1.04 (0.15)	-1.4 (0.08)	1.97 (0.03)	-1.38 (0.09)
P=0.5	**WBV**	-0.78 (0.22)	0.83 (0.20)	-0.96 (0.17)	0.24 (0.41)	0.32 (0.38)	-0.23 (0.41)
	**WMH (tot)**	0.69 (0.24)	0.49 (0.31)	-0.74 (0.23)	-0.13 (0.45)	1.66 (0.05)	1.39 (0.08)

Supplementary Table 4: Results from CORE DATASET 2. T-values (p-values) for a range of KN-BSI whole brain atrophy over scans at months 3, 6, 12 and 24 with respect to natural log of regional WMH (at baseline or month 0) - frontal (F), parietal (P), occipital (O), temporal (T) and basal ganglia + thalami + infratentorial (BGIT); linear regression with confounders for age, sex, education, *APOE*4 burden and five principal components of population structure (including ICV confounder) across diagnostic groups.

	HC					**MCI**					AD				
**KNBSI**	F	P	O	T	BGIT	F	P	O	T	BGIT	F	P	O	T	BGIT
**month3**	2.1 (0.04)	1.8 (0.07)	2.2 (0.03)	1.3 (0.2)	1.3 (0.2)	1.6 (0.1)	1.6 (0.1)	1.5 (0.14)	0.5 (0.65)	1.5 (0.14)	3.1 (0.002)	2.3 (0.02)	3.1 (0.002)	1.2 (0.23)	2.2 (0.03)
**month6**	1.8 (0.07)	2.1 (0.03)	1.8 (0.07)	1.0 (0.3)	2.1 (0.04)	2.4 (0.02)	2.6 (0.01)	2.3 (0.02)	0.87 (0.38)	2.2 (0.03)	2.3 (0.02)	2.6 (0.01)	2.4 (0.02)	0.59 (0.56)	2.4 (0.02)
**month12**	4.5 (9.4e-06)	3.7 (0.0002)	3.8 (0.0001)	2.9 (0.004)	4.5 (8.3e-06)	2.7 (0.008)	2.3 (0.02)	2.6 (0.008)	1.1 (0.27)	2.1 (0.04)	3.2 (0.002)	2.6 (0.01)	2.9 (0.004)	2.0 (0.05)	2.9 (0.004)
**month24**	2.9 (0.003)	2.8 (0.005)	2.6 (0.01)	1.6 (0.2)	2.8 (0.005)	1.5 (0.1)	1.1 (0.27)	1.6 (0.12)	0.4 (0.7)	1.4 (0.16)	0.49 (0.63)	0.03 (0.98)	0.2 (0.87)	-0.45 (0.65)	0.78 (0.44)
